# *Keap1* mutations in lung cancer patients

**DOI:** 10.3892/ol.2013.1427

**Published:** 2013-06-26

**Authors:** HIDEFUMI SASAKI, AYUMI SUZUKI, MASAYUKI SHITARA, KATSUHIRO OKUDA, YU HIKOSAKA, SATORU MORIYAMA, MOTOKI YANO, YOSHITAKA FUJII

**Affiliations:** Department of Oncology, Immunology and Surgery, Nagoya City University Graduate School of Medical Sciences, Nagoya, Aichi 467-8601, Japan

**Keywords:** *Keap1*, NRF2, lung cancer, mutations, adenocarcinoma

## Abstract

Kelch-like ECH-associated protein 1 (Keap1) inhibits nuclear factor erythroid 2-related 2 (NEF2L2; also named NRF2)-induced cytoprotection and has been hypothesized to represent a candidate tumor suppressor. We have previously reported the somatic mutations of the NRF2 gene (*NFE2L2*), however, the correlation between the *Keap1* mutation and the clinicopathological features of lung cancer has not been well investigated. Therefore, in the present study, the *Keap1* mutational status in non-small cell lung cancer (NSCLC) patients was investigated by reverse transcription PCR and direct sequencing. The study included 76 surgically-removed lung cancer cases from patients of the Nagoya City University Hospital in which the *EGFR* and *NFE2L2* mutation status was already established. *Keap1* mutations were identified in 2 (2.6%) adenocarcinoma patients with a history of heavy smoking. These mutations were identified to exist exclusively. The *Keap1* mutation was only detected in patients with advanced adenocarcinoma (4.3%) and the completely exclusive status of this mutation and others, including *EGFR*, *Kas*, *erbB2* and *NRF2L2,* is likely to improve the selection of personalized therapy for lung cancer.

## Introduction

Specific mutations in lung cancer appear to be restricted to specific histologically-defined phenotypes. For example, mutations of tyrosine kinase signaling pathway genes, including *EGFR*(1–3), *ALK*(4), *RET*(5,6) and *erbB2*(7), are common in adenocarcinomas, whereas mutations of the nuclear factor erythroid 2-related 2 (*NEF2L2*; also known as *NRF2*) gene are characteristic of squamous cell carcinoma (8–10).

Under homeostatic conditions, Nrf2 is principally repressed by Kelch-like ECH-associated protein 1 (Keap1), which functions as an intracellular redox sensor, targeting Nrf2 for proteasomal degradation. Under oxidant or xenobiotic stress, Keap1 releases Nrf2, which translocates to the nucleus and activates antioxidant response elements and xenobiotic element genes, resulting in the protein expression of growth factors and receptors, drug-metabolizing enzymes and various transcription factors (11–13). The *Keap1* gene mutation has been previously identified in 3–5% of non-small cell lung cancer (NSCLC) cases (8,14,15), however, the correlation between the mutation status and clinicopathological features was not well defined. We have previously described *NEF2L2* mutation cases (9), and in the present study, the *Keap1* mutation status in 76 surgically-treated NSCLC cases was investigated.

## Patients and methods

### Patients

The current study is retrospective and included data from 76 lung cancer patients who had undergone surgery at the Department of Surgery, Nagoya City University Hospital (Nagoya, China). All tumor samples were immediately frozen and stored at −80°C until assayed. The clinical and pathological characteristics of the 76 lung cancer patients were as follows; 44 cases were at stage I, 11 at stage II and 21 at stages III–IV. The mean patient age was 66.1 years (range, 39–88 years). Among the 76 lung cancer patients, 46 (60.5%) were diagnosed with adenocarcinoma and 27 (35.5%) suffered from squamous cell carcinoma. The study was approved by the ethics board of the Nagoya City University Graduate School of Medicinal Sciences (Nagoya, Chūbu, Japan) and written consent was obtained from all patients.

### PCR for Keap1

Total RNA was extracted from lung cancer tissues using the Isogen kit (Nippon Gene Co., Ltd., Tokyo, Japan), according to the manufacturer’s instructions. The RNA concentration was determined by spectrophotometer and adjusted to a concentration of 200 ng/ml. In 10 cases, the samples were excluded as the number of tumor cells was too low to sufficiently extract tumor RNA. The RNA (1 μg) was reverse transcribed using Superscript II enzyme (Gibco-BRL, Carlsbad, CA, USA) with 0.5 μg oligo(dT)_12–16_ (Amersham Pharmacia Biotech Inc., Piscataway, NJ, USA). The reaction mixture was incubated at 42°C for 50 min and then at 72°C for 15 min. Following this, 1 μl DNA was used for the PCR analyses. PCR was performed using the LA-Taq kit (Takara Bio, Inc., Shiga, Japan) in a 25-μl reaction volume. The primer sequences for the *Keap1* gene kinase domain (exon 2–5) were as follows: forward, 5-AACGGTGCTGTCATGTACCA-3 and reverse, 5-CGCTCTGGCTCATACCTCTC-3 (872 bp). The cycling conditions were an initial denaturation at 94°C for 5 min, followed by 35 cycles at 94°C for 40 sec, 60°C for 40 sec and 72°C for 55 sec. The products were purified by the Qiagen PCR purification kit (Qiagen, Valencia, CA, USA). Amplified cDNAs were separated on 1% agarose gels and the bands were visualized by ethidium bromide. Images were captured under ultraviolet transillumination. These samples were sequenced using the ABI prism 3100 analyzer (Applied Biosystems Japan Ltd., Tokyo, Japan) and analyzed by BLAST. Chromatograms were checked by manual review from forward to reverse.

The *EGFR, erbB2* and *Kras* sequencing methods have previously been described (1,3,7,16).

## Results

### Keap1 gene mutation status in Japanese lung cancer patients

**Figure 1:**
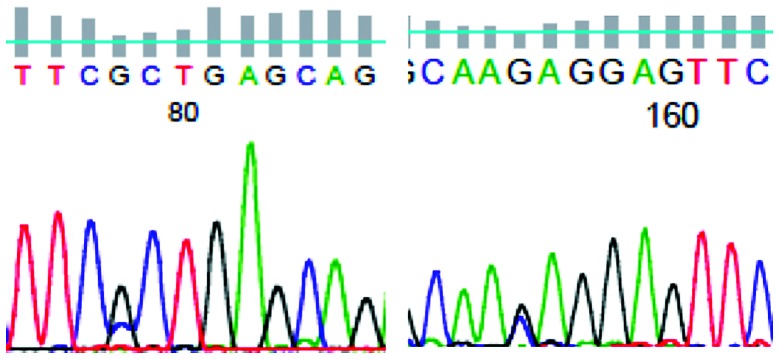
*Keap1* mutation in lung cancer patients: (A) A191P (571 G to C; alanine to proline, stage IIIa) and (B) E218Q (652 G to T, glutamate to glutamine; stage IIb). *Keap1*, kelch-like ECH-associated protein 1.

Of the 76 patients, 19 (25.0%) had *EGFR* mutations within the kinase domain, including 8 exon 19 deletions, 10 L858R and 1 G719S. In addition, 3 patients had *Kras* mutations at codons 12 or 13. The *Keap1* mutation was identified in 2/76 (2.6%) NSCLC patients (Fig. 1); 1 A191P (571 G to C, alanine to proline; stage IIIa) and 1 E218Q (652 G to T, glutamate to glutamine; stage IIb). The two patients were male, had a history of smoking and suffered from adenocarcinoma.

Within these NSCLC cases, the *EGFR*, *Kras, erbB2* and *NRF2* mutations existed exclusively. The survival of the patients with or without the *Keap1* mutations was not shown to be significantly different (log-rank test, P=0.2919).

## Discussion

In the current study, two *Keap1* mutations were identified in 76 Japanese NSCLC patients. The *Keap1* mutation was exclusively identified without *EGFR, erbB2* or *NRF2* mutations. *Keap1* mutations were predominantly identified in patients with a history of heavy smoking and advanced adenocarcinoma. This population was also hypothesized to exhibit a lower incidence of *EGFR* gene mutations (1–3).

The *Keap1* gene is a negative regulator of the cell adaptive response to radical oxidant species and xenobiotics, which is mediated by the NRF2 transcription factor. More recently, a role has emerged for NRF2 in cancer and a number of studies have identified that NRF2 constitutive upregulation is associated with cancer development and progression (17–19). High levels of nuclear NRF2 facilitate cancer cell growth and survival as a result of the transactivation of cytoprotective genes (17–19). Thus, studies on the deregulation of the KEAP1/NRF2 pathway have enhanced our understanding of the molecular mechanisms associated with cancer. We have previously reported that mutations of *NRF2* (*NFE2L2*) were identified in squamous carcinoma cases (9), which was consistent with results shown by additional studies (8,10). In NSCLC, the overexpression of nuclear NRF2 is principally attributable to genetic and epigenetic alterations and the loss of function of its receptor, Keap1 (11,17,20). A previous study demonstrated that low or absent Keap1 expression is common in NSCLC (56%), largely in adenocarcinomas (8). However, the authors identified only one *Keap1* mutation (exon 2–5) in 31 of the tumors examined, including 20 with nuclear NRF2 expression, indicating that the *Keap1* mutation is not the main mechanism of protein loss or reduction. These observations are inconsistent with previous studies reporting *Keap1* mutations in 8 and 19% of two NSCLC cohorts, predominantly with adenocarcinomas (11,17).

*Keap1* mutations are associated with a poor prognosis in individuals with NSCLC (14). In addition, low or absent Keap1 expression is associated with a poor outcome (8). A number of studies have demonstrated that nuclear NRF2 activation promotes cell survival in malignant cells (17,18,21) and may explain the shorter survival of NSCLC. The inactivation of putative tumor suppressor genes affects the growth and progression of tumors. As a mutation of *Keap1* is uncommon, its mechanism in NSCLC remains unknown and may be associated with other Keap1-binding proteins that have antiapoptotic and proliferative functions, including prothymosin a (22).

In the present study, *Keap1* mutations were only identified in patients with adenocarcinoma, but not squamous cell carcinomas. The results indicated that *Keap1* mutations in Japanese individuals with NSCLC are not common, with observed frequencies demonstrated to be even lower compared with our previous *in vitro* analysis in lung cancer cell lines (50%) (11). Present observations revealed that a mutation of the *Keap1* gene as a mechanism of tumorigenesis is unlikely to be associated with the majority of Japanese NSCLC cases. However, the completely exclusive *EGFR*, *NRF2* and *Kras* mutation statuses are likely to be useful for the development of patient-specific therapy for NSCLC. Further studies are required to confirm the mechanisms of *Keap1* mutations to determine the sensitivity or resistance of therapy for lung cancer.
